# Adverse events and adherence to HIV post-exposure prophylaxis: a cohort study at the Korle-Bu Teaching Hospital in Accra, Ghana

**DOI:** 10.1186/s12889-015-1928-6

**Published:** 2015-06-20

**Authors:** Raymond A. Tetteh, Edmund T. Nartey, Margaret Lartey, Aukje K. Mantel-Teeuwisse, Hubert G. M. Leufkens, Priscilla A. Nortey, Alexander N. O. Dodoo

**Affiliations:** Utrecht Institute for Pharmaceutical Sciences, Utrecht University, Universiteitsweg 99, 3584 CG Utrecht, The Netherlands; Pharmacy Department, Korle-Bu Teaching Hospital, P.O. Box KB 77, Korle-Bu, Accra Ghana; World Health Organisation Collaborating Centre for Advocacy and Training in Pharmacovigilance, Centre for Tropical Clinical Pharmacology & Therapeutics, School of Medicine and Dentistry, University of Ghana, P. O. Box GP 4236, Accra, Ghana; Department of Medicine, School of Medicine and Dentistry, University of Ghana, P. O. Box GP 4236, Accra, Ghana; Medicines Evaluation Board, Utrecht, The Netherlands; Department of Epidemiology and Disease Control, School of Public Health, University of Ghana, P.O. Box LG 25, Legon, Accra Ghana

**Keywords:** HIV Post-exposure prophylaxis, Adverse event, Adherence, Cohort event monitoring

## Abstract

**Background:**

There is strong evidence that post-exposure prophylaxis (PEP) with antiretroviral drugs in the timely management of occupational exposures sustained by healthcare workers decreases the risk of HIV infection and PEP is now widely used. Antiretroviral drugs have well documented toxicities and produce adverse events in patients living with HIV/AIDS. In the era of “highly active antiretroviral therapy”, non-adherence to treatment has been closely linked to the occurrence of adverse events in HIV patients and this ultimately influences treatment success but the influence of adverse events on adherence during PEP is less well studied.

**Methods:**

Following the introduction of a HIV post-exposure prophylaxis program in the Korle-Bu Teaching Hospital in January 2005, the incidence of adverse events and adherence were documented in occupationally-exposed healthcare workers (HCWs) and healthcare students (HCSs). Cohort event monitoring was used in following-up on exposed HCWs/HCSs for the two study outcomes; adverse events and adherence. All adverse events reported were grouped by MedDRA system organ classification and then by preferred term according to prophylaxis regimen. Adherence was determined by the completion of prophylaxis schedule. Cox proportional regression analysis was applied to determine the factors associated with the cohort study outcomes. Differences in frequencies were tested using the Chi square test and *p* < 0.05 was considered statistically significant.

**Results:**

A total of 228 exposed HCWs/HCSs were followed up during the study, made up of 101 exposed HCWs/HCSs administered lamivudine/zidovudine (3TC/AZT) for 3 days; 75 exposed HCWs/HCSs administered lamivudine/zidovudine (3TC/AZT) for 28 days; and 52 exposed HCWs/HCSs administered lamivudine/zidovudine/lopinavir-ritonavir (3TC/AZT/LPV-RTV) for 28 days. The frequency of adverse events was 28 % (*n* = 28) in exposed HCWs/HCSs administered 3TC/AZT for 3 days, 91 % (*n* = 68) in exposed HCWs/HCSs administered 3TC/AZT for 28 days and 96 % (*n* = 50) in exposed HCWs/HCSs administered 3TC/AZT/LPV-RTV for 28 days. Nausea was the most commonly reported adverse events in all three regimens. Adherence was complete in all exposed HCWs/HCSs administered 3TC/AZT for 3days, 56 % (*n* = 42) in exposed HCWs/HCSs administered 3TC/AZT for 28 days and 62 % (*n* = 32) in exposed HCWs/HCSs administered 3TC/AZT/LPV-RTV for 28 days. In the Cox regression multi-variate analysis, exposed HCWs/HCSs administered 3TC/AZT for 3 days were 70 % less likely to report adverse events compared with exposed HCWs/HCSs administered 3TC/AZT for 28 days (Adjusted HR = 0.30 [95 % CI, 0.18-0.48], *p* < 0.001). Exposed HCWs/HCSs administered 3TC/AZT for 3 days were 75 % more likely to adhere to the schedule compared with exposed HCWs/HCSs administered 3TC/AZT for 28 days (Adjusted HR = 1.75 [95 % CI, 1.16-2.66], *p* = 0.008).

**Conclusion:**

The intolerance to adverse events was cited as the sole reason for truncating PEP, thereby indicating the need for adequate, appropriate and effective counselling, education, active follow-up (possibly through mobile /phone contact) and management of adverse events. Education on the need to complete PEP schedule (especially for exposed HCWs/HCSs on 28-day schedule) can lead to increased adherence, which is very critical in minimizing the risk of HIV sero-conversion. The present results also indicate that cohort event monitoring could be an effective pharmacovigilance tool in monitoring adverse events in exposed HCWs/HCSs on HIV post-exposure prophylaxis.

## Background

There is evidence that post-exposure prophylaxis (PEP) with antiretroviral drugs in the timely management of occupational exposures sustained by healthcare workers (HCWs) decreases the risk of HIV infection and this type of approach is now widely used [1, 2]. A case control study among HCWs showed that post-exposure use of zidovudine after percutaneous exposure to HIV infected blood was associated with a reduction of the risk of HIV infection by about 81 % [3, 4].

In HIV-infected patients, combination regimens have proven to be superior to monotherapy regimens in reducing viral load [5, 6]. Thus, a combination of drugs with activity at different stages in the viral replication cycle theoretically may offer a greater preventative effect in PEP, particularly for occupational exposures. This is supported by clinical evidence that the use of dual or triple antiretroviral drugs in PEP can prevent sero-conversion by as much as 80 % [7]. However, speed of thought and action is crucial as the window of opportunity to prevent systemic viral dissemination is narrow. Based on these findings, the WHO recommended the use of combination regimens (dual/triple) to prevent HIV sero-conversion in PEP programs which was adopted and adapted by the National HIV/AIDS Control Program (NACP) for use in Ghana [8]. However, the decision to initiate PEP must take into account the potential benefit of preventing infection versus the risk of toxicity from the medications used.

Antiretroviral drugs (ARVs) have well documented toxicities and produce adverse events in patients living with HIV/AIDS. These adverse events have been reported in several studies involving cohorts of patients with incidence rates as high as 54 % being reported in some instances [9, 10]. In the era of “highly active antiretroviral therapy” (HAART), non-adherence to treatment has been closely linked to the occurrence of adverse events in HIV patients and this ultimately influences treatment success [11–14]. HAART is also used for PEP but for a shorter duration of maximally 28 days compared with HIV patients who are generally on life-long medication. However, several studies indicate a higher frequency of reported adverse events in PEP patients who receive multidrug regimens compared with HIV positive patients taking similar or same medication for HIV management [15–22]. This frequency is higher among PEP patients who received triple therapy compared with those who received dual therapy [15, 21] but the rate of discontinuation of PEP was not significantly different between the two PEP therapy groups [21].

With the introduction of HAART, there have been considerable changes in the administered ARV cocktail which comes along with its own adverse events and adherence issues. The use of ARVs in PEP has shifted from the administration of single-drugs like zidovudine to multi-drug regimens involving dual or triple therapy with their associated issues of adverse events and adherence. Very few studies on adverse events and adherence in exposed HCWs/HCSs administered PEP has been conducted in developing countries, hence results from resource-limited settings like the Korle-Bu Teaching Hospital (KBTH) in Accra, Ghana is useful for an effective implementation and education of HCWs on PEP in these settings. Such studies also go a long way to aid program design and national policy in relation to PEP.

The KBTH in Ghana with a workforce of about 1930 HCWs and 3330 healthcare students (HCSs), attends to over 3000 patients daily and also provides HAART services to over 20,000 HIV patients. In 2003, the National AIDS/STI Control Program (NACP) in Ghana provided guidelines on the provision of PEP in all sites that offer clinical care to patients living with HIV including KBTH, one of the four pilot sites for ART delivery at the time. KBTH has since then been offering HAART services to clients regularly.

This study utilizes cohort event monitoring to follow exposed HCWs/HCSs for adverse events and adherence to PEP during a PEP service between January 2005 and December 2010. Cohort-event monitoring (CEM), a prospective and observational cohort study is an adaptation of prescription-event monitoring and it involves actively following up on patients within a defined time-frame for reports of adverse events [23]. CEM employs the technique of actively collecting adverse events reports from patients, mostly by mobile phone calls or, in some cases, direct home visits. Collection of safety data using mobile phones is very appropriate for resource-limited settings like Ghana where the penetration of mobile telephony is high.

## Methods

### Setting

**Fig. 1:**
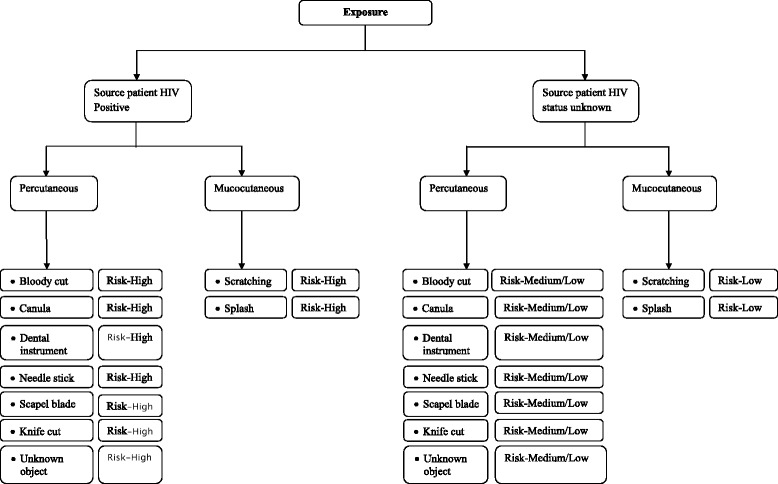
Algorithm used in determining the level of risk of exposures

This study was a prospective cohort analysis of HCWs/HCSs administered PEP at the KBTH in Accra, the premier tertiary referral hospital in Ghana, during the period of January 2005 to December 2010. A team of healthcare professionals made up of medical and pharmacy personnel were responsible for providing PEP service to exposed healthcare workers using an in-house risk assessment system previously described [24] as well as guidelines based on the recommendations of the US Centers for Disease Control and Prevention (CDC) [25]. A risk assessment is done considering the type of injury, the volume of fluid involved, the type of instrument involved (e.g. hollow-bore needle, solid needle etc.) the HIV status of the source patient, the HIV viral load of the source patient and the circumstances surrounding the injury (the depth and extent of injury) as per the national guidelines. This is done on a one-on-one basis for individuals presenting following an exposure. Based on the results of the assessment, a decision is made as to the level of risk of the exposure and the appropriate HIV PEP to be administered according to local guidelines. HCWs/HCSs with exposures assessed as high risk were administered either lamivudine/zidovudine/lopinavir-ritonavir (3TC/AZT/LPV-RTV) (triple therapy) for 28 days or lamivudine/zidovudine (3TC/AZT) (dual therapy) for 28 days. HCWs/HCSs with exposures assessed as medium or low risk were administered either lamivudine/zidovudine (3TC/AZT) (dual therapy) for 28 days or lamivudine/zidovudine (3TC/AZT) for 3 days. However in some exceptional cases, some HCWs/HCSs with exposures assessed as low/medium risk were administered 3TC/AZT/LPV-RTV for 28 days on their insistent request. Figure 1 outlines the algorithm used in the risk assessment of exposures.

### Data collection

The procedures and criteria for the administration of PEP and collection of data (age, gender, HIV status of exposure source, means of exposure, type of exposure, risk assessment, profession, duration of exposure and department where the HCW/HCS belongs) have been described in a previous paper [24]. Exposed HCWs/HCSs were administered either lamivudine/zidovudine (3TC/AZT) (dual therapy) for 3 or 28 days (depending on the outcome of the risk assessment made) or lamivudine/zidovudine/lopinavir-ritonavir (3TC/AZT/LPV-RTV) (triple therapy) for 28 days. Active follow-up on exposed HCWs/HCSs were for two outcomes: a) HIV-testing schedule for possible sero-conversion (at 6 weeks, 3 months and 6 months) and b) adverse events and adherence. Outcome of the follow-up for HIV-testing schedule has been described previously [24].

Active follow-up for adverse events and adherence to prophylaxis schedule were performed by trained research assistants through telephone contact on days 3 and 10 after drug dispensing for those on the 3-day schedule and on days 3, 10, 20, 28 and 35 after drug dispensing for those on the 28 days schedule. In addition to the active follow-up, exposed HCWs/HCSs were asked to report events of medical concern (literally “anything that worries you”) at any time during the follow-up period, noting especially the following signs; fever, rash, lymphadenopathy, dark-coloured urine, sore throat and bruising or bleeding from any part of the body. A structured questionnaire, interview guide was used to collect data from exposed HCWs/HCSs. Event data on adverse events was first recorded according to how the patient described the event (verbatim) and then reviewed qualitatively and coded using the Medical Dictionary for Regulatory Activities (MedDRA, version 13.1) terminology. All adverse events reported were grouped by MedDRA system organ classification (SOC) and then by preferred term (PT) according to prophylaxis regimen. It is important to note that unlike some CEM studies, the presence of adverse events prior to treatment initiation was not undertaken. All adverse events solicited post-treatment were new events that could conceivably be associated with PEP though no formal case-causality assessment was carried out on the individual reports.

Adherence was determined by the completion of prophylaxis schedule assessed during the follow-up period by the use of a questionnaire. Non-adherence was defined as the inability to complete all the 3 days of the prophylaxis schedule for exposed HCWs/HCSs on the 3-day schedule and all the 28 days for exposed HCWs/HCSs on the 28-day schedule as prescribed.

### Data management and analysis

Each exposed HCW/HCS was given a unique study code which was used in data storage and management relating to that HCW/HCS. Data were double entered, cleaned and managed using Microsoft Access (Microsoft Corporation, Redmond, Washington) and analysed using SPSS version 19 (IBM, Armonk, New York). Data were expressed as frequencies and percentages for categorical variables. Differences in frequencies and proportions were tested using the chi square test. The primary outcomes of interest were “adverse events” and “adherence”. Cox proportional regression analysis was applied to determine the factors associated with adverse events/adherence and reported as a crude hazard ratio in the univariate analysis. Variables significantly associated with adverse events or adherence in the univariate analysis (at *p* < 0.10) were adjusted for in the multivariate analysis and reported as an adjusted hazard ratio. *P* < 0.05 was considered statistically significant.

### Ethics

The study was approved by the Ethical and Protocol Review Committee of the University of Ghana Medical School [MS-Et/M.6-P.5.3/2009-10].

## Results

### Exposure information and characteristics of HCWs during reporting and HIV PEP administration

**Fig. 2:**
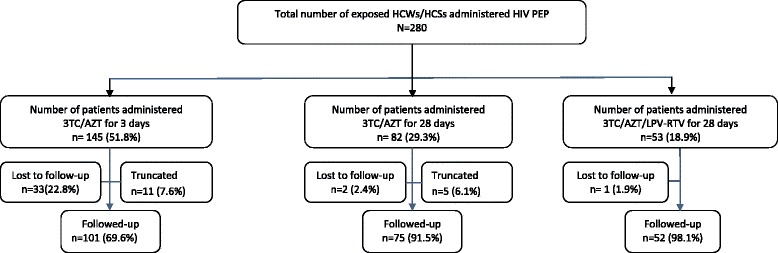
Distribution of exposed HCWs/HCSs administered PEP by regimen

Results from Fig. 2 indicate that out of a total of 280 HCWs/HCs who reported for the PEP service during the study period, 145 (51.8 %) exposed HCWs/HCSs were administered 3TC/AZT for 3 days of which 101 were successfully followed up, 82 (29.3 %) exposed HCWs/HCSs were administered 3TC/AZT for 28 days of which 75 exposed HCWs/HCSs were successfully followed up and 53 (18.9 %) exposed HCWs/HCSs were administered 3TC/AZT/LPV-RTV for 28 days of which 52 exposed HCWs/HCSs were successfully followed up. A total of 16 exposed HCWs/HCSs (11 exposed HCWs/HCSs administered 3TC/AZT for 3 days and 5 exposed HCWs/HCSs administered 3TC/AZT for 28 days) truncated their regimen schedule when the source patient tested HIV negative. The lost to follow-up (LFU) proportion among exposed HCWs/HCSs administered 3TC/AZT for 28 days was significantly lower (*p* < 0.001) compared with those administered 3TC/AZT for 3 days, but not significantly different (*p* = 0. 832) from exposed HCWs/HCSs administered 3TC/AZT/LPV-RTV for 28 days. Over 80 % of the exposed HCWs/HCSs reported their exposure within 24 h and the median time between exposure and reporting was 2.0 h in both HCWs and HCSs. Follow-up for HIV testing was done at 6 weeks, 3 months and 6 months after exposure/HIV PEP administration and none of the HCWs/HCSs followed-up sero-converted.

Table 1 show characteristics of the HCWs/HCSs administered the HIV PEP regimen. The female population constituted 68 % (*n* = 99) of exposed HCWs/HCSs administered 3TC/AZT for 3-days, 50 % (*n* = 41) of exposed HCWs/HCSs administered 3TC/AZT for 28 days and 53 % (*n* = 28) of those administered 3TC/AZT/LPV-RTV for 28 days. The 18–30 years age category represented the largest number of HCWs/HCSs administered all the three PEP regimens (82 % in exposed HCWs/HCSs administered 3TC/AZT for 3-days, 60 % in exposed HCWs/HCSs administered 3TC/AZT for 28 days and 70 % in exposed HCWs/HCSs administered 3TC/AZT/LPV-RTV for 28 days). Whilst risk assessment results for most of the exposed HCWs/HCSs administered 3TC/AZT for 3-days were low (86 %, *n* = 125), the majority of exposed HCWs/HCSs administered 3TC/AZT/LPV-RTV for 28 days risk assessment results was high (83 %, *n* = 44). The majority of exposed HCWs/HCSs administered 3TC/AZT for 28 days risk assessment results were either low risk (35 %, *n* = 29) or medium risk (48 %, *n* = 39). A vast majority of the reported exposures in general were percutaneous (93 %, *n* = 260) with needle stick injuries being the most reported means of exposure (85 %, *n* = 237).

**Table 1:** Baseline characteristics of 280 HCWs/HCSs reporting occupational exposures and administered HIV post-exposure prophylaxis at an urban teaching hospital in Accra, Ghana, 2005–2010

	3TC/AZT-3 days	3TC/AZT-28 days	3TC/AZT/LPV-RTV −28 days
	*N* = 145	*N* = 82	*N* = 53
	n, %^1^	n, %^1^	n, %^1^
Gender			
Male	46 (31.7)	41 (50.0)	25 (47.2)
Female	99 (68.3)	41 (50.0)	28 (52.8)
Age category (yrs)			
18–30	119 (82.1)	49 (59.8)	37 (69.8)
31–40	21 (14.5)	24 (29.3)	10 (18.9)
41–50	3 (2.1)	5 (6.1)	5 (9.4)
>50	2 (1.4)	4 (4.9)	1 (1.9)
Outcome of risk assessment of exposure			
Low	125 (86.2)	29 (35.4)	5 (9.4)
Medium	20 (13.8)	39 (47.6)	4 (7.6)
High	-	14 (17.1)	44 (83.0)
Profession			
Medical Doctors	40 (27.6)	27 (32.9)	22 (41.5)
Nurses	64 (44.1)	26 (31.7)	19 (35.8)
Laboratory Staff	4 (2.8)	8 (9.8)	5 (9.4)
Ward Attendants	13 (9.0)	13 (15.9)	4 (7.5)
Healthcare Students	24 (16.6)	8 (9.8)	3 (5.7)
Type of exposure			
Mucocutaneous	7 (4.8)	3 (3.7)	10 (18.9)
Percutaneous	138 (95.2)	79 (96.3)	43 (81.1)
Year of exposure			
2005	26 (17.9)	14 (17.1)	12 (22.6)
2006	30 (20.7)	10 (12.2)	4 (7.5)
2007	23 (15.9)	15 (18.3)	10 (18.9)
2008	24 (16.6)	16 (19.5)	6 (11.3)
2009	27 (18.6)	15 (18.3)	15 (28.3)
2010	15 (10.3)	12 (14.6)	6 (11.3)
Means of exposure			
Bloody cut	1 (0.7)	1 (1,2)	1 (1.9)
Canula	4 (2.8)	2 (2.4)	16 (30.2)
Dental instrument	1 (0.7)	-	-
Needle stick	133 (91.7)	77 (93.9)	27 (50.9)
Scalpel blade	-	1 (1.2)	2 (3.8)
Scratching	1 (0.7)	-	-
Knife cut	1 (0.7)	-	-
Splash	2 (1.4)	1 (1.2)	7 (12.2)
Unknown object	2 (1.4)	-	-

^1^% are column percentages within each super row; 3TC = lamivudine; AZT = zidovudine; LPV-RTV = lopinavir-ritonavir

### Reporting of adverse events

Out of a total of 228 HCWs/HCSs administered HIV PEP and followed up, 146 (64 %) reported at least one adverse event made up of three spontaneous reports and 143 reports from active surveillance. The proportion of those who reported at least one adverse event was significantly higher (*p* < 0.001) in exposed HCWs/HCSs administered 3TC/AZT for 28 days (*n* = 68, 91 %) compared with exposed HCWs/HCSs administered 3TC/AZT for 3 days (*n* = 28, 28 %) but not significantly different (*p* = 0.236) from exposed HCWs/HCSs administered 3TC/AZT/LPV-RTV for 28 days (*n* = 50, 96 %).

Table 2 shows the Cox regression analyses to determine factors associated with the occurrence of adverse events. In the univariate analysis, gender, profession, age category and type of exposure were not associated with the reporting of adverse events. The outcome of the risk assessment done prior to drug administration (low, medium or high) was associated with adverse events in the univariate but not associated when adjusted for other variables in the multivariate analysis. The duration of drug regimen administered was associated with the reporting of adverse events in both the univariate and multivariate analysis. For exposed HCWs/HCSs administered the same drug regimen (3TC/AZT ) for different durations, those administered 3TC/AZT for 3 days were 70 % less likely to report an adverse event compared with those administered 3TC/AZT for 28 days (Adjusted HR = 0.30 [95 % CI, 0.18-0.48], *p* < 0.001) (Table 2). There was no significant difference (*p* = 0.817) in the reporting of adverse events between exposed HCWs/HCSs administered 3TC/AZT for 28 days and exposed HCWs/HCSs administered 3TC/AZT/LPV-RTV for 28 days in the multivariate analysis.

**Table 2:** Factors associated with reported adverse events in 228 exposed HCWs/HCSs on HIV post-exposure prophylaxis

Characteristic	Adverse events status^1^		Crude hazard ratio [95 % CI]	*p*-value	Adjusted hazard ratio [95 % CI]	*p*-value
	Present	Absent				
Drug regimen						
3TC/AZT −28 days	68	7	1.00		1.00	
3TC/AZT-3 days	28	73	0.31 [0.20–0.48]	<0.001	0.30 [0.18–0.48]	<0.001
3TC/AZT/LPV-RTV-28 days	50	2	1.06 [0.74–1.53]	0.752	1.06 [0.65–1.72]	0.817
Gender						-
Female	81	50	1.00			
Male	65	32	1.08 [0.78–1.50]	0.629		
Type of exposure						-
Percutaneous	131	80	1.00			
Mucocutaneous	15	2	1.42 [0.83–2.43]	0.197		
Age category (yrs)						-
18–30	101	60	1.00			
31–40	32	18	1.02 [0.69–1.52]	0.921		
41–50	8	2	1.28 [0.62–2.62]	0.508		
>51	5	2	1.14 [0.46–2.80]	0.777		
Risk assessment						
Low	51	63	1.00		1.00	
Medium	40	16	1.60 [1.06–2.42]	0.027	0.95 [0.61–1.49]	0.885
High	55	3	2.12 [1.45–3.10]	<0.001	0.97 [0.57–1.66]	0.922
Profession						-
Nurses	51	31	1.00			
HCS	14	10	0.94 [0.52–1.69]	0.832		
Laboratory Staff	15	1	1.51 [0.85–2.68]	0.162		
Medical Doctors	46	31	0.96 [0.65–1.43]	0.843		
Ward Attendants	20	9	1.11 [0.66–1.86]	0.695		

^1^N = 228, exposed HCWs/HCSs lost to follow (n = 36) and exposed HCWs/HCSs who truncated their schedule due to source patient testing HIV negative (n = 16) were excluded; 3TC = lamivudine; AZT = zidovudine; LPV-RTV = lopinavir-ritonavir; CI = confidence interval

### Description of reported adverse events

The most frequently observed type of adverse event reported in all the three PEP regimens was gastrointestinal in nature (Table 3). Nausea, weakness, malaise and dizziness (17 %, 10 %, 6 % and 6 %, respectively) were the four most-commonly reported adverse events in exposed HCWs/HCSs administered 3TC/AZT for 3 days. Exposed HCWs/HCSs on 3TC/AZT for 28 days reported nausea (63 %), weakness (37 %), fatigue (28 %) and dizziness (27 %) as the four most reported adverse events. On the other hand, nausea, diarrhoea, vomiting and weakness (71 %, 65 %, 35 % and 31 %, respectively) were the four most commonly reported adverse events in exposed HCWs/HCSs administered 3TC/AZT/LPV-RTV for 28-days. Nausea was the most commonly reported adverse events in all three regimens. There were a total of five reports of rashes-two reports each in exposed HCWs/HCSs administered 3TC/AZT for 3 days and 28 days and one report in an exposed HCW administered 3TC/AZT/LPV-RTV for 28 days (Table 3).

**Table 3:** Preferred term within system organ classification of reported adverse events of 228 exposed HCWs/HCSs on HIV post-exposure prophylaxis

Number of adverse events reported by preferred term within system organ classification (n, %^1^)	Total	3TC/AZT-3 days	3TC/AZT-28 days	33TC/AZT/LPV-RTV-28 days
	*N* = 228	*N* = 101	*N* = 75	*N* = 52
		n, %^1, 2^	n, %^1, 2^	n, %^1, 2^
Gastrointestinal (*n* = 122, 53.5)				
Nausea	101 (44.3)	17 (16.8)	47 (62.7)	37 (71.2)
Diarrhoea	38 (16.7)	3 (3.0)	1 (1.3)	34 (65.4)
Vomiting	24 (10.5)	1 (1.0)	5 (6.7)	18 (34.6)
Loss of appetite	14 (6.1)	3 (3.0)	6 (8.0)	5 (9.6)
Abdominal pains	14 (6.1)	2 (2.0)	5 (6.7)	7 (13.5)
Anorexia	5 (2.2)	-	2 (2.7)	3 (5.8)
Dehydration	1 (0.4)	-	1 (1.3)	-
Bitter mouth	1 (0.4)	1 (1.0)	-	-
Constipation	1 (0.4)	-	1 (1.3)	-
Excessive flatulence	1 (0.4)	-	-	1 (1.9)
Abdominal discomfort	1 (0.4)	-	1 (1.3)	-
Hyper-salivation	1 (0.4)	-	-	1 (1.9)
Sore throat	1 (0.4)	-	-	1 (1.9)
Excessive spitting	1 (0.4)	-	1 (1.3)	-
Hunger pain	1 (0.4)	-	1 (1.3)	-
Systematic signs & symptoms (*n* = 106, 46.5)				
Weakness	54 (23.7)	10 (9.9)	28 (37.3)	16 (30.8)
Malaise	37 (16.2)	6 (5.9)	17 (22.7)	14 (26.9)
Dizziness	30 (13.2)	6 (5.9)	20 (26.7)	4 (7.7)
Fatigue	28 (12.3)	2 (2.0)	21 (28.0)	5 (9.6)
Feverish	5 (2.2)	-	2 (2.7)	3 (5.8)
General body pains	2 (0.9)	-	2 (2.7)	-
Neurological system (*n* = 42, 18.4)				
Headache	27 (11.8)	3 (3.0)	19 (25.3)	5 (9.6)
Restlessness	8 (3.5)	2 (2.0)	4 (5.3)	2 (3.8)
Insomnia	7 (3.1)	1 (1.0)	4 (5.3)	2 (3.8)
Drowsiness	5 (2.2)	1 (1.0)	1 (1.3)	3 (5.8)
Depression	1 (0.4)	-	1 (1.3)	-
Skin (*n* = 9, 3.9)				
Rashes	5 (2.2)	2 (2.0)	2 (2.7)	1 (1.9)
Itching	2 (0.9)	2 (2.0)	-	-
Alopecia	1 (0.4)	-	1 (1.3)	-
Dark pigmentation of finger & toe nails	1 (0.4)	-	-	1 (1.9)
Central/peripheral nervous system (*n* = 4, 1.8)				
Eye pain	1 (0.4)	-	1 (1.3)	-
Leg pains	1 (0.4)	-	1 (1.3)	-
Neck pains	1 (0.4)	-	-	1 (1.9)
Pain in feet	1 (0.4)	-	-	1 (1.9)
Red eye	1 (0.4)	-	-	1 (1.9)
Hepatitis (*n* = 2, 0.9)				
Yellow eyes	1 (0.4)	-	1 (1.3)	-
Jaundice	1 (0.4)	-	-	1 (1.9)
Cardiac (*n* = 1, 0.4)				
Tightness in chest	1 (0.4)	-	1 (1.3)	-
Reproductive/gynaecological (*n* = 1, 0.4)				
Spontaneous abortion	1 (0.4)	-	-	1 (1.9)
Bleeding	1 (0.4)	-	-	1 (1.9)

^1^Percentages may add up to >100

^2^% are percentages within each drug column; 3TC = lamivudine; AZT = zidovudine; LPV-RTV = lopinavir-ritonavir

There were three spontaneous reports of adverse events which ended up in hospitalisation but all recovered. The first was an exposed female HCW of 26 years administered 3TC/AZT/LPV-RTV for 28 days who reported of dizziness, excessive flatulence, headache, nausea, vomiting, restlessness and dark pigmentation of finger & toe nails (which later came off) and was hospitalised for three days. The second case which resulted in hospitalisation for seven days involved a 29-year old exposed pregnant female HCW (in the first trimester of pregnancy) who reported of abdominal pains, profuse diarrhoea, fatigue, nausea and bleeding which later resulted in spontaneous abortion. The third case involved an exposed female HCW of 42 years who was administered 3TC/AZT for 28 days and reported of fatigue, headache and weakness which according to her resulted in a “near death” experience. She was hospitalised for three days. These three serious adverse events all involving females have been reported to the National Pharmacovigilance Centre at Ghana’s Food and Drugs Authority.

### Adherence to prophylaxis schedule

Complete adherence to PEP schedule was 77 % (*n* = 175) in this study. All the 53 PEP exposed HCWs/HCSs who defaulted in their 28-days prophylaxis schedule did so within 14 days of medication initiation. None of the exposed HCWs/HCSs on the 3-day schedule defaulted in medication adherence. Comparing the different PEP regimens administered, adherence to the prophylaxis schedule was significantly higher (*p* < 0.001) in exposed HCWs/HCSs administered 3TC/AZT for 3-days (100 %, *n* = 101) compared with exposed HCWs/HCSs administered 3TC/AZT for 28-days (56.0 %, *n* = 42) and exposed HCWs/HCSs administered 3TC/AZT/LPV-RTV for 28-days (61.5 %, *n* = 32). However, there was no statistical difference (*p* = 0. 534) in adherence proportion between exposed HCWs/HCSs administered 3TC/AZT for 28-days and exposed HCWs/HCSs administered 3TC/AZT/LPV-RTV for 28-days.

Table 4 shows the factors associated with adherence in both the univariate and multivariate Cox regression analysis. Gender, profession, outcome of risk assessment, type of exposure and age category was not associated with adherence in the univariate analysis. For the same drug regimen exposed HCWs/HCSs administered 3TC/AZT for 3-days were 75 % more likely to adhere to the schedule compared with exposed HCWs/HCSs administered 3TC/AZT for 28-days (Adjusted HR = 1.75 [95 % CI, 1.16-2.66], *p* = 0.008). There was no statistical significant difference in terms of adherence between exposed HCWs/HCSs administered 3TC/AZT for 28 days and exposed HCWs/HCSs administered 3TC/AZT/LPV-RTV for 28 days (*p* = 0.660).

**Table 4:** Factors associated with adherence in exposed HCWs/HCSs on HIV post-exposure prophylaxis

Characteristic	Adherence Status^1^		Crude hazard ratio [95 % CI]	*p*-value	Adjusted hazard ratio [95 % CI]	*p*-value
	Adhered	Defaulted				
Drug regimen						
3TC/AZT −28 days	42	33	1.00		1.00	
3TC/AZT-3 days	101	0	1.79 [1.25–2.56]	0.002	1.75 [1.16–2.66]	0.008
3TC/AZT/LPV-RTV −28 days	32	20	1.10 [0.69–1.74]	0.688	1.02 [0.56–1.88]	0.938
Gender						
Female	102	29	1.00			
Male	73	24	0.97 [0.72–1.31]	0.824	-	-
Age range (years)						
18–30	129	32	1.00			
31–40	34	16	0.85 [0.58–1.24]	0.395		
41–50	6	4	0.75 [0.33–1.70]	0.489		
>51	6	1	1.07 [0.47–2.43]	0.872	-	-
Risk assessment						
Low	103	11	1.00		1.00	
Medium	36	20	0.71 [0.49–1.04]	0.079	0.91 [0.60–1.39]	0.672
High	36	22	0.69 [0.47–1.00]	0.052	1.05 [0.56–2.00]	0.871
Type of exposure						
Percutaneous	165	46	1.00			
Mucocutaneous	10	7	0.75 [0.40–1.42]	0.382	-	-
Profession						
Nurses	65	17	1.00			
HCS	19	5	1.00 [0.60–1.67]	0.996		
Laboratory Staff	9	7	0.71 [0.35–1.43]	0.335		
Medical Doctors	59	18	0.97 [0.68–1.38]	0.850		
Ward Attendants	23	6	1.00 [0.62–1.61]	0.998	-	-

^1^N = 228, exposed HCWs/HCSs lost to follow (*n* = 36) and exposed HCWs/HCSs who truncated their schedule due to source patient testing HIV negative (*n* = 16) were excluded; 3TC = lamivudine; AZT = zidovudine; LPV-RTV = lopinavir-ritonavir; CI = confidence interval

In a separate analysis to determine the association between the two study outcomes (report of adverse events and adherence), exposed HCWs/HCSs who never reported of adverse events were 57 % more likely to adhere to their medication schedule compared with exposed HCWs/HCSs who reported of at least one adverse event (HR = 1.57 [95 % CI, 1.17-2.11], *p* = 0.003). All the 53 exposed HCWs/HCSs who did not adhere completely to their PEP medication schedule cited adverse events as their reason for non-adherence.

## Discussion

The findings provide insight into the occurrence of adverse events and adherence to medication schedule in HIV negative HCWs/HCSs who presented for PEP at the KBTH between January 2005 and December 2010. The present results show that adverse events are very common (1 in 10) in exposed HCWs/HCSs undergoing different regimens, more so with the 28-day regimens. Again, it showed that serious adverse events leading to hospitalization were common, occurring in this case at a frequency higher than 1 in 100 (3 serious adverse events in 228 exposed HCWs/HCSs). All the exposed HCWs/HCSs who reported serious adverse events were females and were on 28 days regimen schedule. The importance of the duration of treatment on adverse events and adherence is starkly demonstrated by the fact that adverse events were low and adherence excellent in the 3 days regimen schedule whilst adverse events were more common and adherence varied between 56 and 62 % in the 28 days regimen schedule.

In this study exposed HCWs/HCSs administered PEP were allowed to describe their complaints in their own context before the use of the system organ class (SOC) classification to group the adverse events reported. In general, these exposed HCWs/HCSs administered PEP were not on other medications of note and therefore related these events to the anti-retroviral medications administered. The events reported did not exist prior to initiation of therapy. The present results indicate an overall adverse event frequency of 64 %, which is lower than the results of other studies where the frequency of adverse events was reported to be between 70 and 76 % [15, 18, 26]. However the reported high adverse events frequency of 96 % in exposed HCWs/HCSs administered 3TC/AZT/LPV-RTV (triple therapy) for 28 days is comparable to reported adverse events frequency of also 96 % in PEP patients administered the same regimen of 3TC/AZT/LPV-RTV for 28 days in sexual assault survivors [27].

As expected, the adverse events reported were consistent with the product profiles (Summary of Product Characteristics-SmPC) of the PEP medications in use. The most frequently cited symptoms were gastrointestinal in nature; vomiting, diarrhoea, and nausea followed by neurological symptoms like headache, weakness and fatigue which is similar to results from other PEP studies [15, 18, 19, 28].

The study recorded three serious adverse events – all cases of hospitalisations following initiation of PEP. Worth noting was the case of dark pigmentation (hyper pigmentation) of finger and toe nails reported by a patient on 3TC/AZT/LPV-RTV for 28 days which incidentally has been reported in some other studies as a cutaneous adverse reaction of zidovudine (AZT), mostly in patients on chronic treatment of HIV [28]. The case of spontaneous miscarriage was recorded in a patient in the first trimester of pregnancy. The three anti-retroviral drugs involved (3TC/AZT/LPV-RTV) are classified as pregnancy category “C” and were used in as the benefits of the combination appeared to outweigh any potential risks. Whilst other causes for spontaneous abortion could not be ruled out in this patient, the occurrence of this event following intake of 3TC/AZT/LPV-RTV necessitates constant vigilance on the relationship between pregnancy outcomes and intake of the combination in order to generate enough evidence to support its continual use in pregnancy, especially during the first trimester whether used for PEP or for HAART. Both cases were also associated with severe diarrhoea and vomiting and the subjects were rehydrated, stabilized and discharged, with appropriate counselling to continue and complete the course of 28 days since their risk assessment were rated high. The third case of serious adverse event of fatigue, headache and weakness involving an exposed female HCW of 42 years was also stabilized and discharged (after three days hospitalisation) with appropriate counselling to continue and complete the course of 28 days.

This study could not establish any association between the report of at least one adverse events and factors such as gender, age, profession, outcome of risk assessment and type of exposure in the multivariate analysis although other studies have associated adverse events with gender [17]. The present results showed that for the same type of drug regimen (dual therapy) administered as PEP, exposed HCWs/HCSs on the longer schedule (28 days) experienced more adverse than and were more likely to be non-adherent to treatment than those on shorter schedules. Even though other studies have shown an association of more frequent adverse events with triple therapy compared with dual therapy [1, 15, 21, 22], the present results rather indicate that the association may be due to the duration of therapy since there was no difference in frequency of adverse events when both the dual therapy (3TC/AZT) and triple therapy (3TC/AZT/LPV-RTV) were administered for 28 days. The association of longer duration of prophylaxis (28-days) to the reporting of adverse events may be due to the time to onset of adverse events as studies [15, 18] have shown adverse events to begin to appear from the fourth day of drug administration.

Results from this study indicate an overall adherence rate of 77 %. Although this adherence rate is far higher than reported in some studies [15, 26], it is comparable to other reports [19, 29]. Similar to the adverse events reportage, shorter therapy schedules of 3 days were associated with better (excellent in this study) adherence compared with the longer duration of 28 days. It has been shown that knowledge and proper communication of expected side effects of ARVs in patients leads to increased rate of adherence to the treatment [30]. The possibility of receiving either dual or triple therapy for 28 days depends on the outcome of the risk assessment which leaves no choice for the exposed HCW/HCS. Education and counselling are therefore very important in ensuring maximum adherence. The intolerability to adverse events was cited as the sole reason for truncating PEP, thereby indicating the need for adequate, appropriate and effective counselling, education, active follow-up (possibly through mobile/phone contact) and management of adverse events. Education on the need to complete PEP schedule (especially for exposed HCWs/HCSs on 28-day schedule) can lead to increased adherence which is very critical in preventing HIV sero-conversion. This may also reduce anxieties associated with the injuries. In addition, the need for monitoring and managing of adverse events may encourage completion of PEP schedule by HCWs who are exposed [31].

The use of the CEM technique in this study has led to the successful monitoring of adverse events and adherence to PEP schedule. This study has demonstrated that is it possible to effectively conduct an active safety monitoring of ARVs used in PEP in resource limited settings like the KBTH in Accra, Ghana. The safety data obtained from this CEM study are consistent with the safety profile expected for the medicines concerned. Since the tendency to passively/spontaneously report adverse events and adherence in HIV negative exposed HCWs/HCSs on PEP is generally low, CEM will become a key pharmacovigilance method in resource-limited settings due to its ability to aid in rapid identification of signals and to effectively compare regimen and the effect of treatment type and duration on both adverse events and adherence.

Most of the exposed HCWs/HCSs were willing to be followed up by phone and loss to follow up was reasonably low compared to other studies in these settings. Although the inability to follow exposed HCWs/HCSs administered PEP beyond 6 months to monitor possible long term toxicities is a limitation, monitoring of long term toxicities was not the focus of the current study. Since laboratory tests for liver enzymes, full blood count, etc. were not conducted, it is not known to what extent the medicines might have caused abnormalities which could only be detected via laboratory testing. However the relatively shorter period of 28 days given for PEP does not permit extrapolation of data to the identification of possible long-term toxicities that might have occurred if these products had been taken for long periods. The results cannot therefore be extrapolated to verify or refute known HAART induced toxicities like hepatitis or zidovudine-induced anaemia [32] which occur during the initial months of therapy in HIV-positive patients. Despite this, intensive laboratory monitoring during the period PEP should be encouraged and facilitated to identify all toxicities.

Although the results of this study cannot be necessarily generalized across HIV/AIDS treatment sites in Ghana or in West Africa and other resource-limited settings, the incidence of adverse events and non-adherence provides an insight into what to expect in such settings and this information could be used for patient education and the education of HCWs as PEP provision is scaled up. This study has shown the importance of focused cohort studies in generating important safety information in resource-limited settings where the spontaneous reporting ADR systems yield very little data and where healthcare systems e.g. absence of electronic health records and weak laboratory infrastructure, do not permit collection of safety data in any systematic manner. A possible limitation of the current study is the in-house medication adherence metrics used. Although medication adherence metrics are available including the one validated by Morisky et al. [33], none was used in this HIV PEP situation where exposed HCWs/HCSs may take the prophylactic medication for a maximum of 28 days. There is therefore the need to develop specific tools for measuring medication adherence in PEP whether short (3 days) or long (28 days) treatment periods are chosen.

## Conclusions

In conclusion, it is possible to use active follow-up method via mobile phone calls to monitor both adverse events and adherence to prophylaxis regimen in HIV-negative PEP subjects. The study showed that adverse events to PEP are very common and could be severe or serious requiring hospitalization for management in some cases and that the nature and frequency of adverse events collected in the current study were consistent with available information on the use of those antiretroviral drugs. Much emphasis should be attached to follow-ups in order to advise and act promptly when issues arise. This study also indicated the need for education of exposed HCWs/HCSs on the importance of complete adherence to HIV PEP in order to minimize the risk of HIV sero-conversion.

## Abbreviations

HCW: Healthcare worker
HCS: Healthcare student
3TC: Lamivudine
AZT: Zidovudine
LPV-RTV: Lopinavir-ritonavir
PEP: Post-exposure prophylaxis
PT: Preferred term
SOC: System organ classification
MedDRA: Medical Dictionary for Regulatory Activities
